# Idiopathic pulmonary fibrosis with bronchogenic carcinoma

**DOI:** 10.4103/0970-2113.48899

**Published:** 2009

**Authors:** Girija Nair, Shivani Swami, Ankur Mehta

**Affiliations:** *Department of Chest Disease, Pad. Dr D.Y. Patil College and Hospital, Nerul, Navi Mumbai, India*

**Keywords:** Interstitial pulmonary fibrosis, lung cancer, wood smoke

## Abstract

Idiopathic pulmonary fibrosis is essentially a benign disease. We present a case of 65-year-old female patient who presented with left-sided chest pain and breathlessness. Her CT thorax revealed idiopathic pulmonary fibrosis along with a left-sided pleural-based mass. Biopsy of the mass revealed a squamous cell carcinoma. She was exposed to home wood smoke while cooking on the *chulla* for many years, which was possibly responsible for both the diseases.

## INTRODUCTION

Idiopathic pulmonary fibrosis has been considered a benign disease. It presents with breathlessness and cough. A higher incidence of lung cancer has been described in patients of idiopathic pulmonary fibrosis.^1^–^4^ We are presenting such a case.

## CASE HISTORY

A 65-year-old lady presented with gradually progressing breathlessness for last six months, and left-sided dull aching continuous chest pain that did not radiate. She also complained of occasional dry cough. There was a history of chronic exposure to home wood smoke while cooking over the *chulla* since 50 years. On examination, there was pallor and grade II clubbing. Pulse was 84/min, blood pressure 130/80 mm Hg, and respiratory rate 28/min. No past history of pulmonary tuberculosis or diabetes was present. Also, no history of any drug intake in the past was present.

Breath sounds were decreased at the left infrascapular area and crepitations were present in bilateral basal areas. Cardiac examination was normal, She was conscious, and her liver and spleen were not palpable. No abdominal lump was felt. Chest X-ray revealed left lower lobe homogenous opacity with well-defined margins.

**Figure 1:**
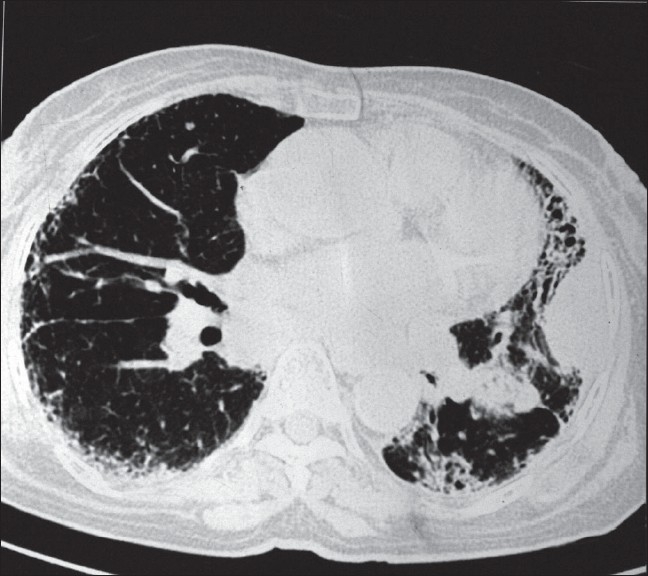
HRCT thorax showing honeycombing with bilateral reticular lesions and a left-sided pleural-based mass

Her complete blood count was within normal limit, fasting blood sugar was 90 mg/dl, and serum creatinine was 0.9 mg/dl. Three consecutive sputum smears were negative for acid fast bacilli (AFB). Sputum cytology for malignant cells (three samples) was also negative. CT thorax (plain and contrast enhanced) revealed bilateral extensive fibrosis with honey combing and a pleural-based mass lesion in the left lung [Figure 1]. Also, a lytic lesion was present in second dorsal vertebral body. On fine needle aspiration cytology (FNAC), the left-sided mass felt hard and gritty, and revealed squamous cell carcinoma.

## DISCUSSION

Idiopathic pulmonary fibrosis is a progressive fibrosing inflammatory disease of the lung of unknown etiology.^3^ Most patients present with dyspnea, fatigue, clubbing, crepitations, and interstitial radiological changes suggestive of fibrosis. On lung biopsy, there may be inflammation and fibrosis distal to terminal bronchioles.

Idiopathic pulmonary fibrosis is usually considered as being benign; however, there are reports of an increased incidence of lung cancer in patients having idiopathic pulmonary fibrosis.^1^,^2^

The etiology of idiopathic interstitial fibrosis is not well known. It may be due to genetic factors as it may occur in many members of a family. There may be a genetic basis in lung fibrogenic response.^2^ Environmental exposure to asbestos, cytotoxic drugs, or Epstein Barr virus infection may also play a role. Idiopathic pulmonary fibrosis is also seen following mycoplasma infection. Interstitial fibrosis is also noted in patients of rheumatoid arthritis and other connective tissue diseases. Interstitial fibrosis has also been described in people having home heating with wood.^3^,^4^

Lung cancer was found in 9.8% patients of idiopathic pulmonary fibrosis in a large study of 205 patients. The relative risk for male and female smokers was 14.2 and 6.7%, respectively. The authors concluded that there is an increased risk of lung cancers in patients of idiopathic pulmonary fibrosis which is not wholly accounted for by age, sex, or smoking habit. The distribution of histological types was similar to that found in lung cancer without pulmonary fibrosis. In their study, large opacities suggestive of lung cancer were present at first hospital visit in 20% of patients who had cancer.^4^

Our patient came with history of increasing breathlessness of 5–6 months duration along with on and off cough. Her chest pain was of 2–3 weeks duration. At this stage, her CT thorax revealed extensive idiopathic pulmonary fibrosis, a left-sided pleural-based mass lesion, and invasion of vertebral bodies. FNAC revealed squamous cell carcinoma of the lung. Her chest pain could have been due to vertebral body involvement by the squamous cell carcinoma or its metastasis.

She was exposed to home wood fire (*chulla*) while cooking, for over 50 years. This long wood smoke exposure could have induced idiopathic pulmonary fibrosis, squamous metaplastic changes in the bronchial epithelium, and ultimately lung cancer. Kitchen smoke due to wood fire cooking without proper exhaust system or chimney can cause respiratory symptoms and watering of eyes in people (usually woman). This type of cooking is common in the Indian villages.

The higher incidence of lung cancers in patients of idiopathic pulmonary fibrosis could be due to the common environmental factor – atmospheric pollution. Home wood smoke during cooking thus may cause interstitial fibrosis as well as cancers of the lung.
